# Antidromic Atrioventricular Reentry Tachycardia with Wolff Parkinson White Syndrome: A Rare Beast

**DOI:** 10.7759/cureus.2642

**Published:** 2018-05-17

**Authors:** Rizwan Ali, Arooj Tahir, Muhammad Nadeem, Mohammed I Shakhatreh, Brett Faulknier

**Affiliations:** 1 Internal Medicine, Rapides Regional Hospital, Alexandria, USA; 2 Medicine, St Francis Medical Center; 3 Cardiology Department, Charleston Area Medical Center/west Virginia University

**Keywords:** wpw, avrt

## Abstract

Orthodromic atrioventricular reentrant tachycardia (AVRT) is the second-most-common form of supraventricular tachycardia (SVT) and is inducible in approximately 55% of individuals with Wolff Parkinson White (WPW) syndrome. Antidromic AVRT, where the accessory atrioventricular connection is used as the antegrade limb and the atrioventricular node serves as the retrograde limb of the circuit, has been clinically documented in less than 5% of patients with WPW syndrome and may be induced in less than 10% of these WPW cases in the electrophysiology laboratory. Left lateral pathways are considered more frequent and septal locations are less common when associated with antidromic AVRT.

We report a case of 21-year-old male with a history of WPW syndrome who had undergone a prior electrophysiology study in 2010 at an outlying facility, documenting an anteroseptal accessory pathway near the His bundle along with an unsuccessful attempt at radiofrequency ablation at that time. No supraventricular tachycardia was induced at that previous study. The surface electrocardiogram (ECG), at this time, was consistent with the anteroseptal WPW pattern. The patient now presented with a complaint of intermittent palpitations with no definitive trigger. He also described a recent syncopal episode while walking inside his home. His physical exam and all lab work were within normal limits for his age. He underwent a repeat electrophysiology (EP) study where the baseline PR interval was 62 milliseconds and the QRS duration was 172 milliseconds in a pre-excited pattern. There was found to be an antegrade-only conducting accessory pathway at the anteroseptal region near the His bundle. Antegrade AVRT was induced with a single ventricular extra stimulus while on 2 mcg/min isoproterenol. Cryoablation was performed in a position slightly posterior to the His bundle, which successfully resolved the accessory pathway conduction. First-degree atrioventricular (AV) block was noted in the sinus rhythm with a PR interval of 226 milliseconds post-cryoablation. There was no recurrence of accessory pathway conduction on follow-up ECG 24 hours post-cryoablation.

Antidromic AVRT is a very rare finding in WPW syndrome during an EP study. Catheter ablation is the treatment of choice for patients who have symptomatic WPW syndrome. Catheter ablation can be especially challenging when the accessory pathway is in close proximity to the normal conduction pathways. The prognostic significance of inducible antidromic AVRT is controversial in asymptomatic patients and limited data indicate it may be a poor prognostic sign in children. In adults, the prognostic significance is not well-established. Cryoablation is an option for the ablation of accessory pathways that are close to the normal conduction pathways. “Cryomapping” is designed to have precise ablation and to reassure the absence of complications.

## Introduction

The WPW is an accessory pathway (AP) mediated tachycardia occurring in patients with ventricular pre-excitation on a 12-lead electrocardiogram (ECG). Ventricular pre-excitation occurs in 0.1 to 3.1 out of 1000 people [1]. Ventricular pre-excitation is the activation of the ventricular myocardium by an atrial impulse earlier than would be expected with normal AV conduction. A delta wave is seen on ECG, which represents the activation of the ventricle by an AP before activation by the normal conducting system. APs are formed from the incomplete segmentation of the embryologic cardiac tube and the formation of a fibrotic atrioventricular ring during fetal cardiac development. The most common type of pathway is AV, formed by myocardial tissue connecting the atrium and ventricle [2]. Most pathways are epicardial. AV pathways can “manifest,” which means that they conduct antegradely from the atrium to the ventricle and result in pre-excitation that can be seen on the surface ECG. AV pathways can be “inapparent,” which means pre-excitation is not seen on the surface ECG or concealed because normal AV conduction activates the ventricle faster than the AP. Inapparent AV pathways conduct only retrograde from the ventricle to the atrium and are clinically relevant only when they participate in a tachycardia [3].

## Case presentation

**Figure 1:**
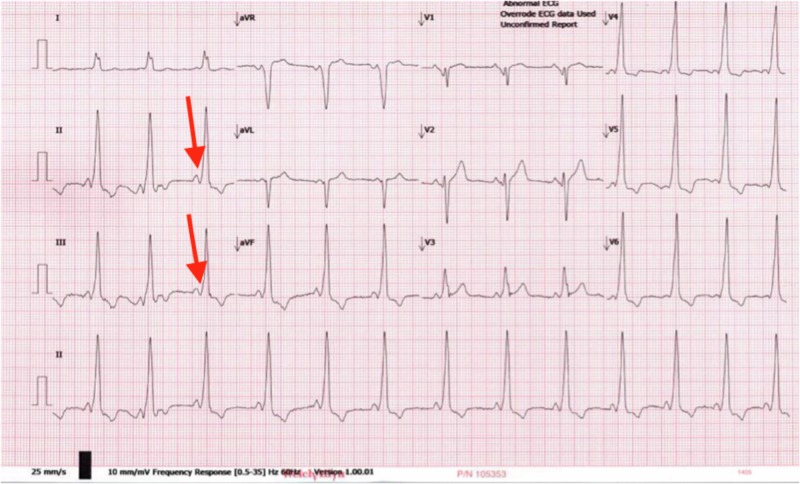
ECG before procedure The arrows are showing delta waves and the short PR interval. ECG: electrocardiogram

We report a case of a 21-year-old male with a history of WPW syndrome who had undergone a prior electrophysiology study in 2010 at an outlying facility, documenting an anteroseptal accessory pathway near the His bundle along with an unsuccessful attempt at radiofrequency ablation at that time. No supraventricular tachycardia was induced at that previous study. The surface ECG, at this time, was consistent with the anteroseptal WPW pattern, as shown in Figure 1.

**Figure 2:**
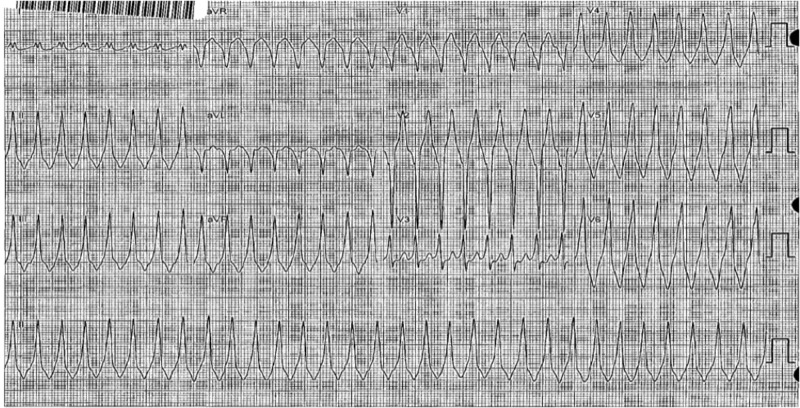
Ventricular tachycardia during the EP study EP: electrophysiology

**Figure 3:**
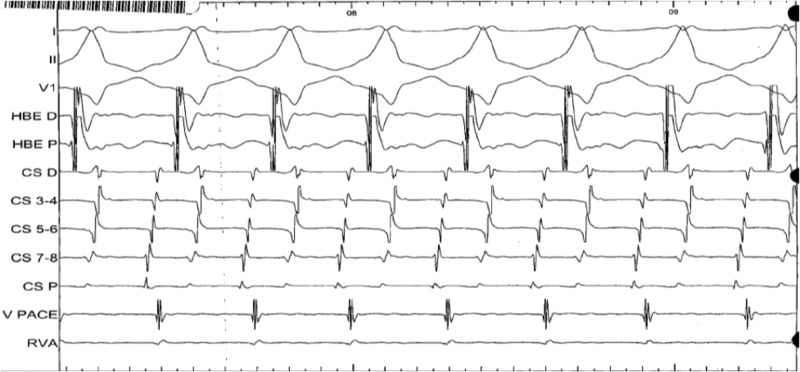
Intracardiac ECG during the electrophysiology study ECG: electrocardiogram

The patient now presented with a complaint of intermittent palpitations with no definitive trigger. He also described a recent syncopal episode while walking inside his home. His physical exam and all lab work were within normal limits for his age. He underwent a repeat EP study where the baseline PR interval was 62 milliseconds and the QRS duration was 172 milliseconds in a pre-excited pattern. There was found to be an antegrade-only conducting accessory pathway at the anteroseptal region near the His bundle. Antegrade AVRT was induced with a single ventricular extra stimulus while on 2 mcg/min of isoproterenol, as shown in Figures 2-3.

**Figure 4:**
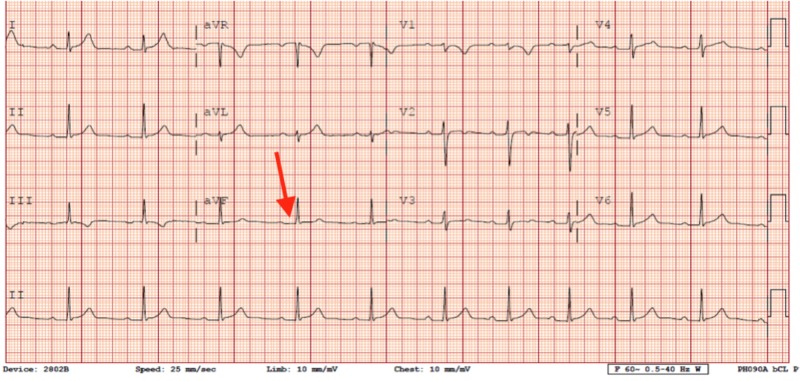
ECG after ablation of accessory pathway Arrow showing a first-degree AV block, There is no delta wave and a short PR interval. AV: atrioventricular

Cryoablation was performed in a position slightly posterior to the His bundle, which successfully resolved the accessory pathway conduction. A first-degree AV block was noted in a sinus rhythm with a PR interval of 226 milliseconds post-cryoablation. There was no recurrence of accessory pathway conduction on follow-up ECG 24 hours post-cryoablation, as shown in Figure 4.

## Discussion

**Figure 5:**
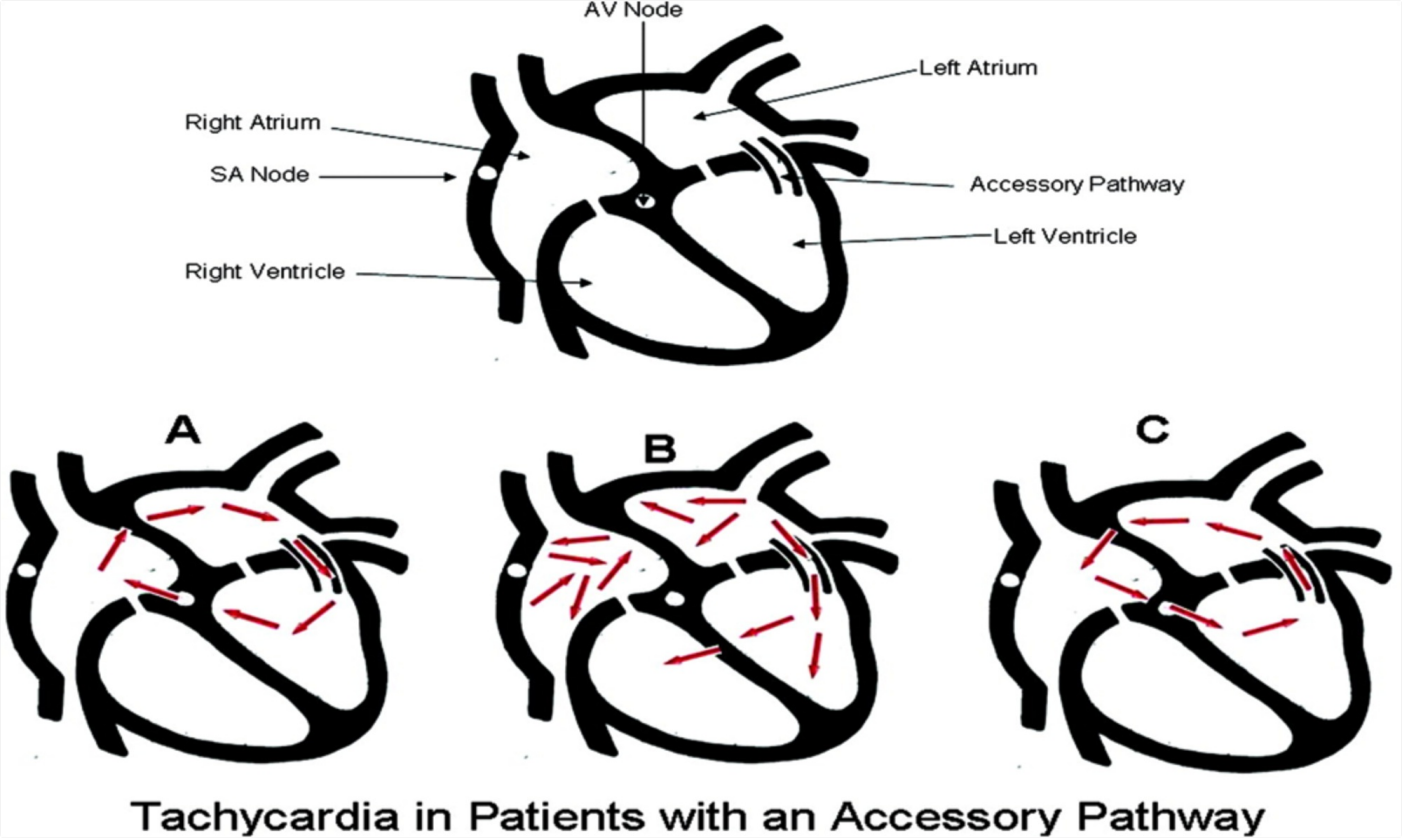
The schematic diagram of different types of arrhythmias in WPW A. Antidromic AVRT, B. AF, C. orthodromic AVRT Abbreviations: AV: atrioventricular, SA: sinoatrial, AVRT: atrioventricular reentery tachycardia, WPW: Wolff Parkinson White

Types of arrhythmias in WPW are AVRT (80%). There are two type of AVRT, orthodromic (90 to 95%) vs. antidromic (5%), other types are atrial fibrillation (AF) (10% to 30%), atrial flutter, and ventricular tachycardia/ventricular fibrillation [4-7]. The different types of arrhythmias are shown in Figure 5.

Antidromic AVRT is an uncommon finding in WPW syndrome. Catheter ablation is the treatment of choice and can be especially challenging once the accessory pathway is in close proximity to the normal conducting pathways.

The prognostic significance of inducible antidromic AVRT is controversial in asymptomatic patients. In adults, the prognostic significance is not well-established in asymptomatic patients. Cryoablation is an option for the ablation of accessory pathways that are close to the normal conduction pathways. Cryomapping is designed to do precise ablation and to reassure the absence of complications.

## Conclusions

Antidromic AVRT is a very rare finding in WPW syndrome during an EP study. Catheter ablation is the treatment of choice for patients who have symptomatic WPW syndrome. Catheter ablation can be especially challenging when the accessory pathway is in close proximity to the normal conduction pathways. Cryoablation is an option for the ablation of accessory pathways that are close to the normal conduction pathways. “Cryomapping” is designed to do precise ablation and to reassure the absence of complications.
